# Supplementary material for: Efficacy of Wheelchair Skills Training Program in Enhancing Sitting Balance and Pulmonary Function in Chronic Tetraplegic Patients: A Randomized Controlled Study
Source: Medicina (Kaunas). 2023 Sep 6;59(9):1610. doi: 10.3390/medicina59091610 (PMC10536794; doi:10.3390/medicina59091610)
Supplement: Supplementary file 1 [file medicina-59-01610-s001.zip › medicina-2563192-supplementary.pdf]

Sitting Balance Subscale:

1. Sitting with back unsupported but feet supported on the floor or on a foot stool

Administration of item: The participant should be seated in a standard-height chair without armrests. The participant should be positioned on the chair so that his/her back is not touching the back of the chair and his/her lower extremities have 90° of flexion in the hips, knees, and ankles. If the participant cannot achieve a full neutral pelvis due to an orthopedic condition (e.g. lumbar stenosis, fusion of vertebrae), have the participant sit as upright as possible and score appropriately.

Instruction to participant: Please sit up as straight as you can, with a slight arch in your low back and with your arms folded or resting in your lap for 2 min.

Scoring:

4. Able to sit with a neutral pelvis (neither anteriorly nor posteriorly tilted) independently, 2 min

3. Able to sit 2 min with posterior pelvic tilt, independently

2. Able to sit 30 s with posterior pelvic tilt, with supervision

1. Only able to sit with posterior pelvic tilt, 10 to 29 s, with supervision

0. Unable to sit without support 10 s

2. Seated forward reach

Administration of item: The participant should be seated in a standard-height chair without armrests, leaning against the back of the chair, with his/her sacrum approximately 3 in from the back of the chair, so that his/her back is on an 80° incline. The participant should have 90° of flexion in the knees and ankles, with both feet resting on the floor. A meter stick will be held by another examiner at the height of the participant's shoulder. The participant will flex one shoulder to 90°; the other upper extremity may rest in the participant's lap, but cannot provide support. The ulnar styloid process should be used as a bony landmark for measurement. If the participant is unable to flex either upper extremity to 90°, then both upper extremities can rest in the participant's lap but may not be used for support. In this case, the acromion can be used as the bony landmark for measurement. At no point should the participant touch or rest against the meter stick.

Instruction to participant: Please raise your preferred arm up to the height of your shoulder. Reach forward as far as possible, and then return to an upright position without using your hands for support. Do not twist your trunk as you reach.

Scoring: Upper extremity used (please circle): Right Left

4. Able to reach forward 15 in independently
3. Able to reach forward 10 to 15 in independently
2. Able to reach forward 5 to 10 in but needs supervision
1. Reaches forward 5 in and needs supervision
0. Loses balance when trying, requires physical assistance

3. Seated lateral reach

Administration of item: The participant should begin while seated in the same position as for the seated forward reach test, in a chair without armrests. Prior to reaching laterally, the participant should sit upright so that his/her trunk is no longer touching the back of the chair. When reaching to the right, the participant should abduct the right shoulder to 90°, and the ulnar styloid process should be used as the bony landmark for measurement. The left upper extremity may rest in the participant's lap, but cannot be used for support. If the participant is unable to abduct the shoulder to 90°, then the acromion may be used as the bony landmark. Repeat with the left upper extremity. Score each upper extremity separately. The participant's hips may come up on the opposite side of the reach.

Instruction to participant: Please raise one arm up to the height of your shoulder. Reach out to the right as far as possible and return to the middle. Wait 5 s, then reach out to the left as far as possible and return to the middle. Do not twist your trunk while you reach, and keep your feet flat on the floor.

Scoring: Please mark score in the box provided

Right	Left

4. Able to reach >6 in independently

3. Able to reach 2 to 6 in with supervision

2. Able to reach 2 in with supervision

1. Able to turn head in direction of reach and uses contralateral limb in lap to assist during reach with supervision

0. Loses balance when trying, requires physical assistance

4. Pick up/touch an object from the floor from a seated position

Administration of the item: The participant should begin while seated in the same position as for the seated forward reach test in a chair without arm rests. A 12- to 16-oz plastic cup should be placed on the floor between the participant’s feet. Any strategy may be used to pick up the cup, including the use of 2 hands on the cup. If the participant is unable to pick up the cup because of impaired hand function, he/she may just touch the cup.

Instruction to participant: Please pick up the cup placed in front of your feet any way you like. Try to use your arms for balance as little as possible.

Scoring:

4. Able to pick up/touch cup independently without using arms to maintain balance.

3. Unable to pick up/touch the cup but comes within 1 to 2 in of the cup and keeps balance independently without using arms

2. Able to pick up/touch the cup independently but uses arms for support

1. Reaches halfway to cup and needs supervision while trying

0. Loses balance when trying, requires physical assistance to keep from falling

5. Scooting forward in a chair

Administration of the item: The participant should be seated in a standard-height chair without arm rests with his/her feet in contact with the floor, sitting back as far as possible in the chair so that his/her back is against the backrest. In order to move forward, the participant may scoot the buttocks forward either unilaterally or bilaterally. The participant should not push against the back of the chair to slide the buttocks forward. The examiner may demonstrate segmentally moving each buttock forward.

Instruction to participant: Please move your bottom forward to the edge of the chair, using your arms if necessary. Do not push against the back of the chair.

Scoring

4. Able to move one buttock forward at a time without assistance, without upper extremities

3. Able to move both buttocks forward simultaneously, with or without upper extremities

2. Able to lift buttocks off of chair, but unable to move forward, with or without upper extremities

1. Requires minimal assistance to lift buttocks and move forward, with or without upper extremities

0. Requires moderate to maximal assistance to lift buttocks and move forward, with or without upper extremities

6. Wheelchair-to-chair transfers

Administration of item: The participant should be seated in a standard-height chair without armrests. Arrange a standard-height/standard-width manual wheelchair with a solid seat and no back cushions (use chair size to keep hip and knee flexion roughly at 90° perpendicular to each other for a stand or squat pivot/lateral transfer). The participant may use a sliding board if necessary, but cannot score higher than 2. The left armrests and footrests may be removed prior by the examiner prior to the transfer.

Instruction to participant: Please transfer from the chair you are sitting in, to the wheelchair next to you, using your hands as little as possible. Then, when you are ready, please transfer back into the other chair. You may use a sliding board if you need one.

Scoring:

4. Able to independently perform a stand pivot/stand step transfer without use of hands

3. Able to perform a stand pivot/stand step transfer with use of hands as a guide, with no weight bearing through upper extremities, requires supervision

2. Able to perform a squat pivot/lateral transfer with use of upper-extremity weight bearing without a sliding board

1. Able to perform a squat pivot/lateral transfer with use of upper-extremity weight bearing with a sliding board 0. Needs physical assist with or without a sliding board.

7. Support surface displacement while seated in a wheelchair

Administration of the item: The participant should be seated in a standard-height/standard-width manual wheelchair, as described in item 7. The participant holds a 12-in-diameter inflatable beach ball with both hands and/or wrists, while his/her feet are supported on wheelchair footrests. The brake on the left wheel should be locked. Facing the participant, the examiner contacts the top of the propulsion rim on the right side of the wheelchair with his/her left hand, while guarding the individual with his/her right arm. The chair is then turned one eighth of a circle (or 45°) forward in 1 s by pulling the hand down toward the floor. After a balance response is made or once the participant is returned to an upright sitting posture, the examiner returns the propulsion rim rapidly back (45° in 1 s) to the starting position. The trunk is unsupported during this test, and the participant is not allowed to bear weight through the hands on the lap during the test.

Instruction to participant: Hold the ball with both hands and raise it as high as you can. Keep your trunk still while I turn your chair. Try not to lean against the back of the chair.

Scoring:

4. Able to raise ball to 90° shoulder flexion with elbows extended and maintain or recover balance during turns in both directions.

3. Able to raise ball 3 in off lap and maintain or recover balance while turning one direction only

2. Keeps hands on ball in lap but does not bear weight through the upper extremities and trunk remains steady during turns in both directions.

1. Keeps hands on ball in lap and upper-extremity weight bearing is used to recover trunk balance during turns in both directions.

0. Unable to sit unsupported for 30 s, unable to attempt or tolerate perturbations.

Program List for Wheelchair Skill Training Program 4.1

Skill Level	Individual Skills
Indoor	Rolls forward 10m
Community	Rolls forward 10m in 30s
Indoor	Rolls backward 5m
Indoor	Turns 90° while moving forward L&R
Indoor	Turns 90° while moving backward L&R
Indoor	Turns 180° in place L&R
Indoor	Maneuvers sideways L&R
Indoor	Gets through hinged door in both directions
Indoor	Reaches 1.5m high object
Indoor	Picks object from floor
Indoor	Relieves weight from buttocks
Indoor	Transfers from WC to bench and back
Community	Folds and unfolds wheelchair
Community	Rolls 100m
Community	Avoids moving obstaclesL&R
Community	Ascends 5° incline
Community	Descends 5° incline
Advanced	Ascends 10° incline
Advanced	Descends 10° incline
Community	Rolls 2m across 5° side-slope L&R
Community	Rolls 2m on soft surface
Community	Gets over 15cm pot-hole
Community	Gets over 2cm threshold
Community	Ascends 5cm level change
Community	Descends 5cm level change
Advanced	Ascends 15cm curb
Advanced	Descends 15cm curb
Advanced	Performs 30s stationary wheelie
Advanced	Turns 180° in place in wheelie position L&R
Advanced	Gets from ground into wheelchair
Advanced	Ascends stairs
Advanced	Descends stairs
